# Divergent estimates of herd‐wide caribou calf survival: Ecological factors and methodological biases

**DOI:** 10.1002/ece3.6553

**Published:** 2020-07-21

**Authors:** E. Hance Ellington, Keith P. Lewis, Erin L. Koen, Eric Vander Wal

**Affiliations:** ^1^ School of Environment and Natural Resources Ohio State University Columbus OH USA; ^2^ Department of Biology Memorial University of Newfoundland St. John’s NF Canada; ^3^ Northwest Atlantic Fisheries Centre Fisheries and Oceans Canada St. John’s NF Canada; ^4^ Wildlife Research and Monitoring Section Ontario Ministry of Natural Resources and Forestry Peterborough ON Canada

**Keywords:** composition surveys, mortality risk, multiple imputation, Newfoundland, survival analysis, woodland caribou (*Rangifer tarandus caribou*)

## Abstract

Population monitoring is a critical part of effective wildlife management, but methods are prone to biases that can hinder our ability to accurately track changes in populations through time. Calf survival plays an important role in ungulate population dynamics and can be monitored using telemetry and herd composition surveys. These methods, however, are susceptible to unrepresentative sampling and violations of the assumption of equal detectability, respectively. Here, we capitalized on 55 herd‐wide estimates of woodland caribou (*Rangifer tarandus caribou*) calf survival in Newfoundland, Canada, using telemetry (*n* = 1,175 calves) and 249 herd‐wide estimates of calf:cow ratios (C:C) using herd composition surveys to investigate these potential biases. These data included 17 herd‐wide estimates replicated from both methods concurrently (*n* = 448 calves and *n* = 17 surveys) which we used to understand which processes and sampling biases contributed to disagreement between estimates of herd‐wide calf survival. We used Cox proportional hazards models to determine whether estimates of calf mortality risk were biased by the date a calf was collared. We also used linear mixed‐effects models to determine whether estimates of C:C ratios were biased by survey date and herd size. We found that calves collared later in the calving season had a higher mortality risk and that C:C tended to be higher for surveys conducted later in the autumn. When we used these relationships to modify estimates of herd‐wide calf survival derived from telemetry and herd composition surveys concurrently, we found that formerly disparate estimates of woodland caribou calf survival now overlapped (within a 95% confidence interval) in a majority of cases. Our case study highlights the potential of under‐appreciated biases to impact our understanding of population dynamics and suggests ways that managers can limit the influence of these biases in the two widely applied methods for estimating herd‐wide survival.

## Graphical abstract

We investigated whether estimates of calf mortality risk and calf:cow ratios were biased by unrepresentative sampling and violations of equal detectability, respectively, using 35 years of caribou monitoring data. We found that caribou calves collared later in the calving season had a higher mortality risk and caribou calf:cow ratios were higher when surveys were conducted later in the autumn. If these biases go undetected, they could impact management.

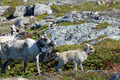

## 1 INTRODUCTION

Population monitoring is fundamental to species conservation and management (IUCN, 2012) and can be used to track the abundance of wildlife populations through time so that managers can adjust management actions accordingly (Gibbs, Snell, & Causton, 1999; Nichols & Williams, 2006). Indeed, many jurisdictions rely on monitoring initiatives to inventory and manage large mammal and big game species (Flather, Knowles, & Brady, 2009; Manitoba Sustainable Development, 2018; Resources Inventory Committee, 2002). Insufficient or inadequate data on population trends can hamper conservation efforts (e.g., Blake & Hedges, 2004). Even long‐term, seemingly well‐designed monitoring programs can lead to poor conservation practices (Gibbs et al., 1999; Karanth et al., 2003). There are numerous biases, most of which we are unaware of, that can threaten our ability to accurately monitor population change through time. It is therefore prudent that we attempt to identify and correct biases in monitoring programs. This is especially important for vulnerable and at‐risk species.

**FIGURE 1:**
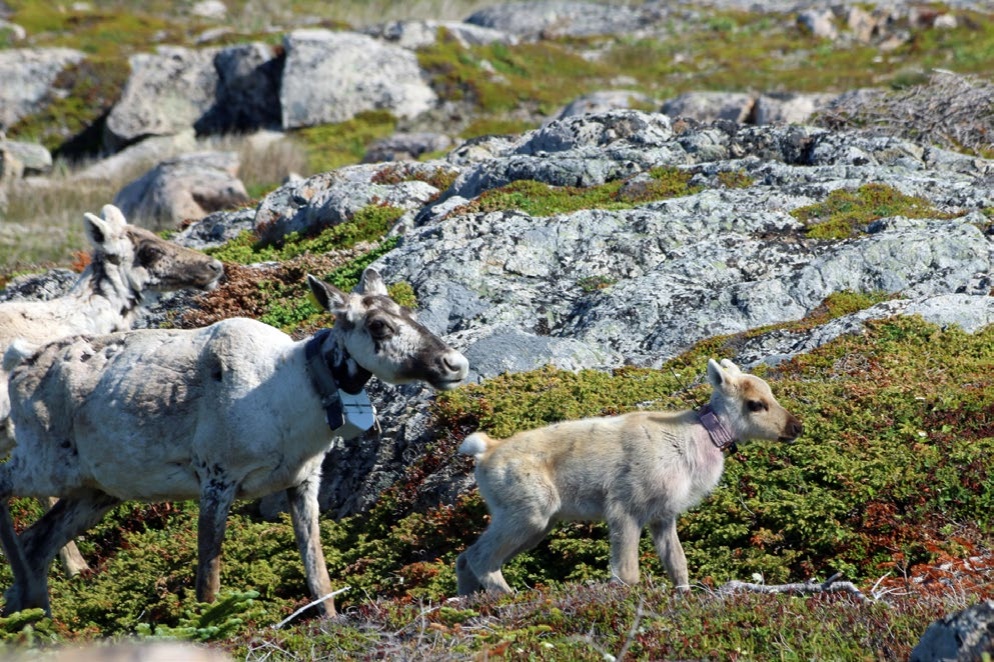
Woodland caribou (*Rangifer tarandus caribou*) cow and calf on Fogo Island, Newfoundland on 28 June 2016 taken by Maegwin Bonar

Caribou (*Rangifer tarandus*; Figure 1) are becoming a global flagship species of concern due to climate and other anthropogenic causes of population decline (Festa‐Bianchet, Ray, Boutin, Côté, & Gunn, 2011; Vors & Boyce, 2009). In Newfoundland, Canada, woodland caribou (*R. t. caribou*) have been monitored nearly continuously for over 35 years, revealing large changes in population abundance that mirror the declines in caribou populations in the circumpolar north (Festa‐Bianchet et al., 2011; Vors & Boyce, 2009). In Newfoundland, woodland caribou population abundance was comparatively low during the 1960s and 1970s, increased rapidly during the 1980s to mid‐1990s, and has since declined sharply (Bastille‐Rousseau, Schaefer, Mahoney, & Murray, 2013). This decline, from approximately 94,000 woodland caribou at its peak to an estimated 31,000 in 2013, led the Committee on the Status of Endangered Wildlife in Canada to designate the woodland caribou population in Newfoundland as “Special Concern” (COSEWIC, 2016).

**FIGURE 2:**
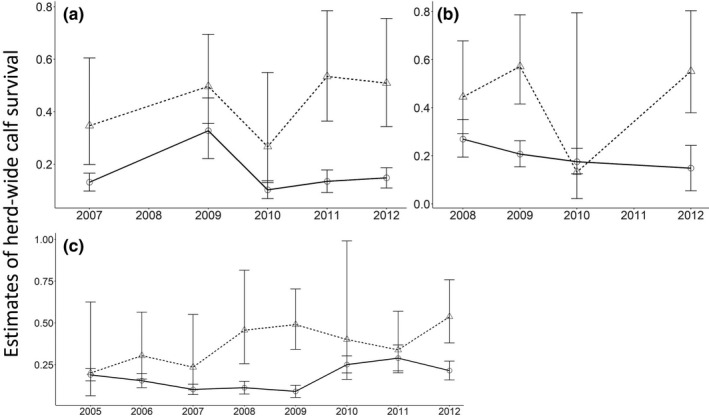
Estimates of herd‐wide woodland caribou (*Rangifer tarandus caribou*) calf survival. Estimates were derived from telemetry (triangle and dashed line) and herd composition survey (circle and solid line) datasets for herds in (a) La Poile, (b) Northern Peninsula, and (c) Middle Ridge during the population decline (2003–2014) in Newfoundland, Canada. Estimates represent unmodified data and demonstrate the lack of congruence in estimates of herd‐wide calf survival from the two data sources

**FIGURE 3:**
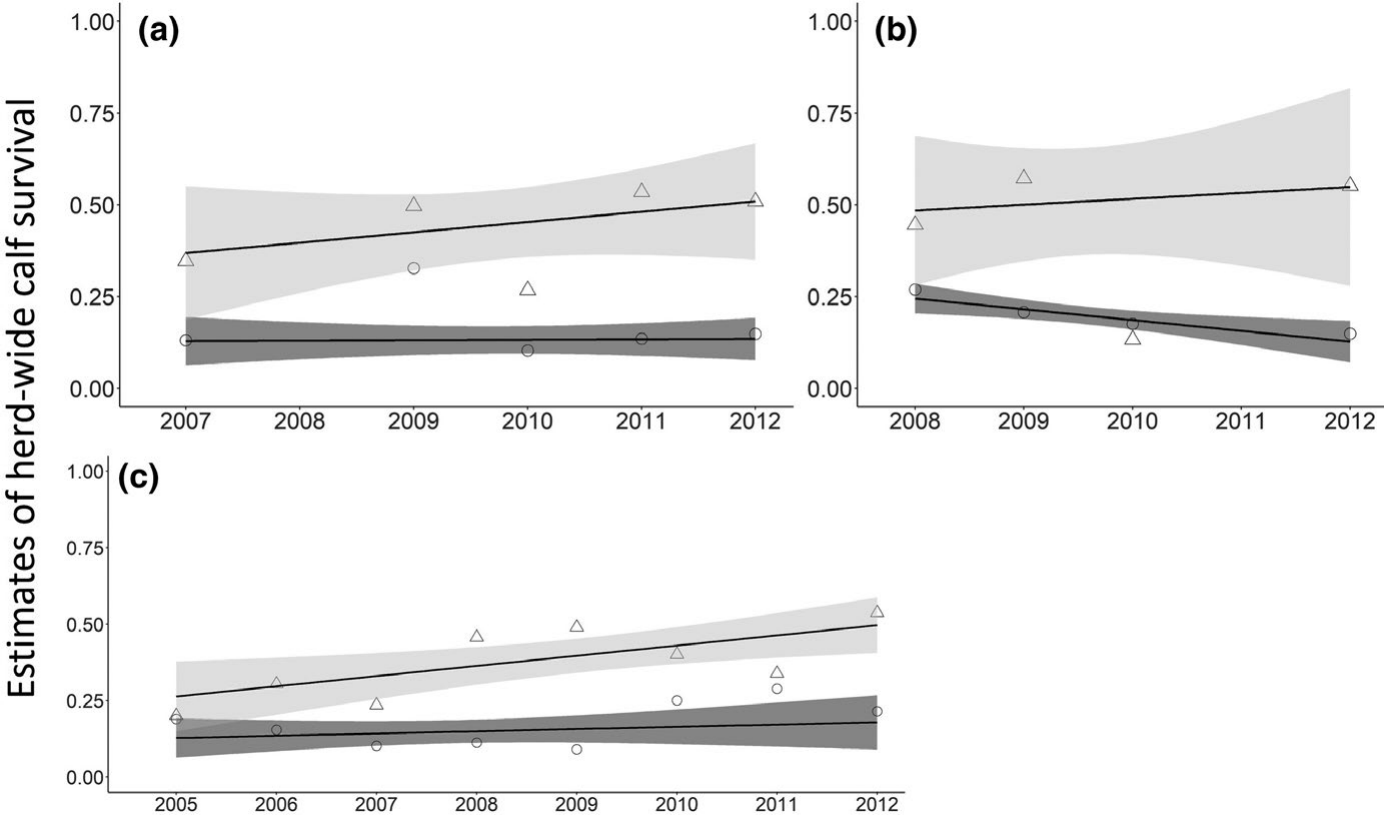
Weighted trend line (using the inverse sample variance) and 85% confidence intervals (for clarity) of estimates of herd‐wide woodland caribou (*Rangifer tarandus caribou*) calf survival. Data were derived from telemetry (triangle and light gray ribbon) and herd composition survey (circle and dark gray ribbon) datasets for herds in (a) La Poile, (b) Northern Peninsula, and (c) Middle Ridge during the population decline (2003–2014) in Newfoundland, Canada. Estimates represent unmodified data and demonstrate the lack of congruence in estimates of herd‐wide calf survival from the two data sources

In an attempt to understand the cause of woodland caribou population declines in Newfoundland, managers estimated calf survival from two independent and occasionally concurrent sets of monitoring data: recruitment rates (calf:cow ratio, hereafter, C:C) from herd composition surveys (e.g., Bender, 2006) and individual estimates of calf survival from telemetry (e.g., Olson, Fuller, Schaller, Lhagvasuren, & Odonkhuu, 2005). As originally interpreted, trends of herd‐wide woodland caribou calf survival in Newfoundland derived from herd composition surveys and telemetry generally agreed during the population growth phase (1979–1997), but appeared to diverge during the population decline phase (2002–2014); the telemetry data suggested a gradual increase in calf survival over time, while the herd composition surveys suggested that C:C remained low (Weir, Morrison, Luther, & Mahoney, 2014; Figures 2 and 3). This discrepancy in estimates of calf survival for the same herds during the same time period suggests that there are biases in the data because we would expect both methods to show the same trend in herd‐wide calf survival through time. These biases would otherwise have gone undetected if we did not have two datasets to compare; these datasets thus allow us the unique opportunity to evaluate biases that could influence management.

Herd composition surveys and telemetry are common methods for estimating calf survival in ungulates, and both can be prone to biases that may lead to erroneous management or conservation actions (Elphick, 2008; Gilbert, Lindberg, Hundertmark, & Person, 2014; Murray, 2006). Calf:cow estimates derived from herd composition surveys depend on the critical assumption that different demographic groups are equally detectable at the time of the survey. Violations to this assumption can arise if: (a) the probability of detection varies among demographic groups (McCorquodale, 2001); (b) if some demographic groups are more likely absent (e.g., variation in the timing of herd aggregation for different demographic groups); or (c) if some demographic groups are present but difficult to numerate or are misclassified due to habitat type (Bonenfant, Gaillard, Klein, & Hamann, 2005; Samuel, Garton, Schlegel, & Carson, 1987) or physical similarities among demographic groups (Citta, Quakenbush, & Taras, 2014). Equal detectability during herd composition surveys of woodland caribou could be affected by survey date if different demographic groups (e.g., cows with calves and cows without calves) aggregate at different times. Similarly, larger herd sizes might hinder the detection of certain demographic groups.

Survival estimates derived from telemetry studies assume that marked individuals represent the whole herd and that the collaring and monitoring processes do not influence survival (Cattet, Boulanger, Stenhouse, Powell, & Reynolds‐Hogland, 2008). Efforts to collar woodland caribou calves might only occur on a few days of the calving season; thus, the distribution of calf collaring dates (typically an index of calf birth date) in a telemetry sample is unlikely to represent the distribution of calf birth dates in the herd. This violation of the assumption of representative sampling (i.e., only monitoring calves born during a few days of the calving season) could generate a biased estimate of survival if woodland caribou calves that were born later in the calving season have a lower probability of survival; indeed, this pattern is evident across several ungulate species (Festa‐Bianchet, 1988; Gaillard, Festa‐Bianchet, Yoccoz, Loison, & Toïgo, 2000). While several studies have examined the potential biases of herd composition surveys (e.g., Caughley, 1974; McCullough, 1994) and survival analyses (e.g., DeCesare, Hebblewhite, Lukacs, & Hervieux, 2016; Gilbert et al., 2014; Murray, 2006), and examined how vital rates themselves might influence age ratios derived from herd composition surveys (Harris, Kauffman, & Mills, 2008), rarely have both methods been evaluated concurrently on the same herd. Violations of these assumptions could lead to the divergent estimates of woodland caribou calf survival that we observed.

**FIGURE 4:**
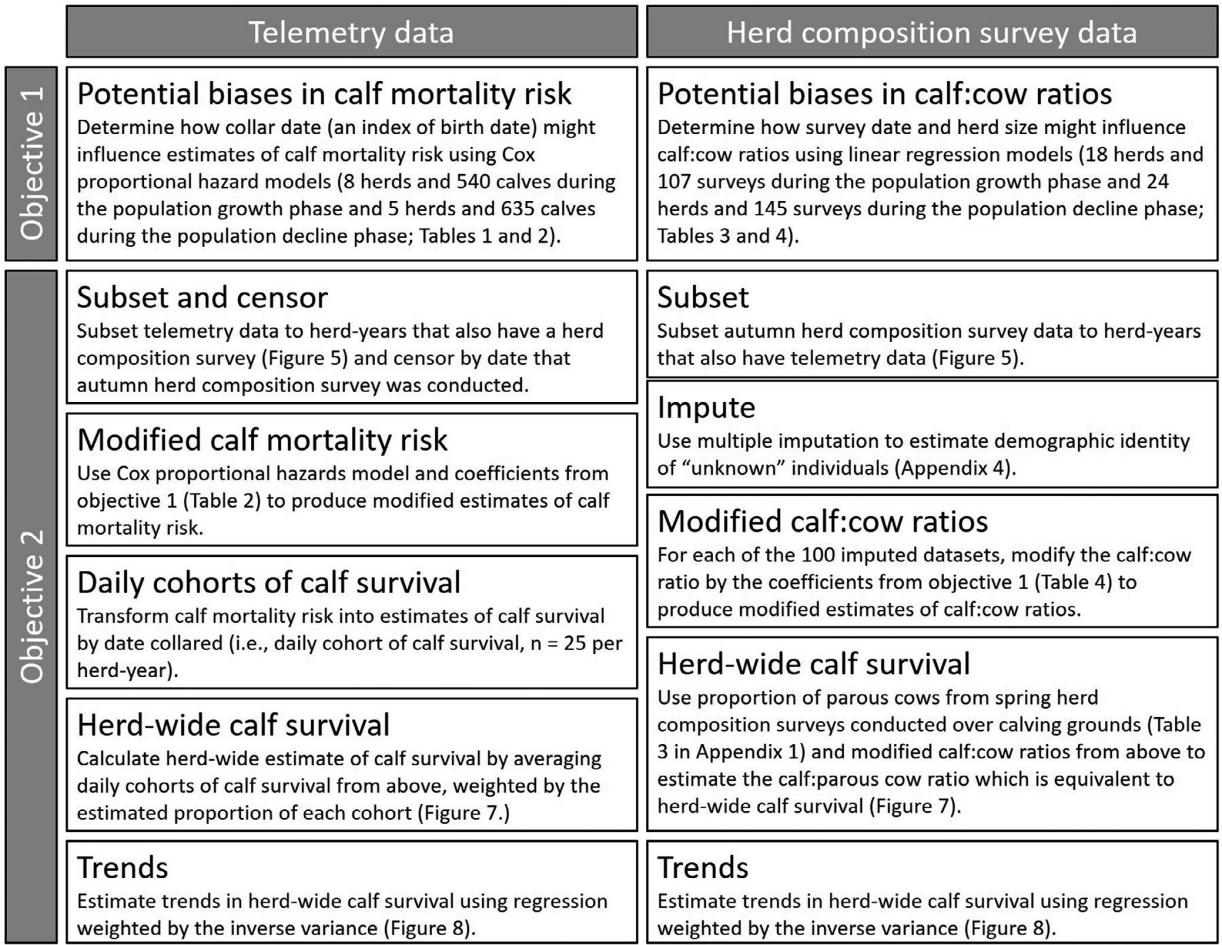
Schematic diagram of our methodology for producing modified estimates of herd‐wide woodland caribou (*Rangifer tarandus caribou*) calf survival and trends from telemetry data (left column) and herd composition survey data (right column) across three herds (La Poile, Northern Peninsula, and Middle Ridge) during the population decline (2003–2014) in Newfoundland, Canada

The goal of our analysis was to identify and attempt to reconcile the methodological challenges that cause divergent estimates of herd‐wide calf survival from herd composition surveys and telemetry datasets, therefore lending increased confidence to potential management and conservation practices. Our first objective was to determine how the date that calves were collared influenced estimates of calf survival and how survey date and herd size influenced C:C across both the population growth and decline phases of woodland caribou in Newfoundland. Our second objective was to determine whether modification of herd‐wide calf survival estimates (based on the relationships identified in the first objective) could reconcile trends in herd‐wide calf survival derived from telemetry and herd composition surveys (Figure 4).

## 2 METHODS

### 2.1 Study area and woodland caribou herds

The island of Newfoundland, Canada, had a maritime climate that was cool year‐round. Dominant land cover consisted of coniferous and mixed forests of balsam fir (*Abies balsamea*), black spruce (*Picea mariana*), and white birch (*Betula papyrifera*), interspersed with lakes, bogs, and barren rock. Woodland caribou in Newfoundland were distributed throughout most of the island in distinct herds. Every year, female woodland caribou on the island move to herd‐specific calving grounds during spring and summer. Major predators of woodland caribou calves on the island included American black bear (*Ursus americanus*), Canada lynx (*Lynx canadensis*), and coyote (*Canis latrans*) (Bastille‐Rousseau et al., 2016; Mahoney et al., 2016).

**FIGURE 5:**
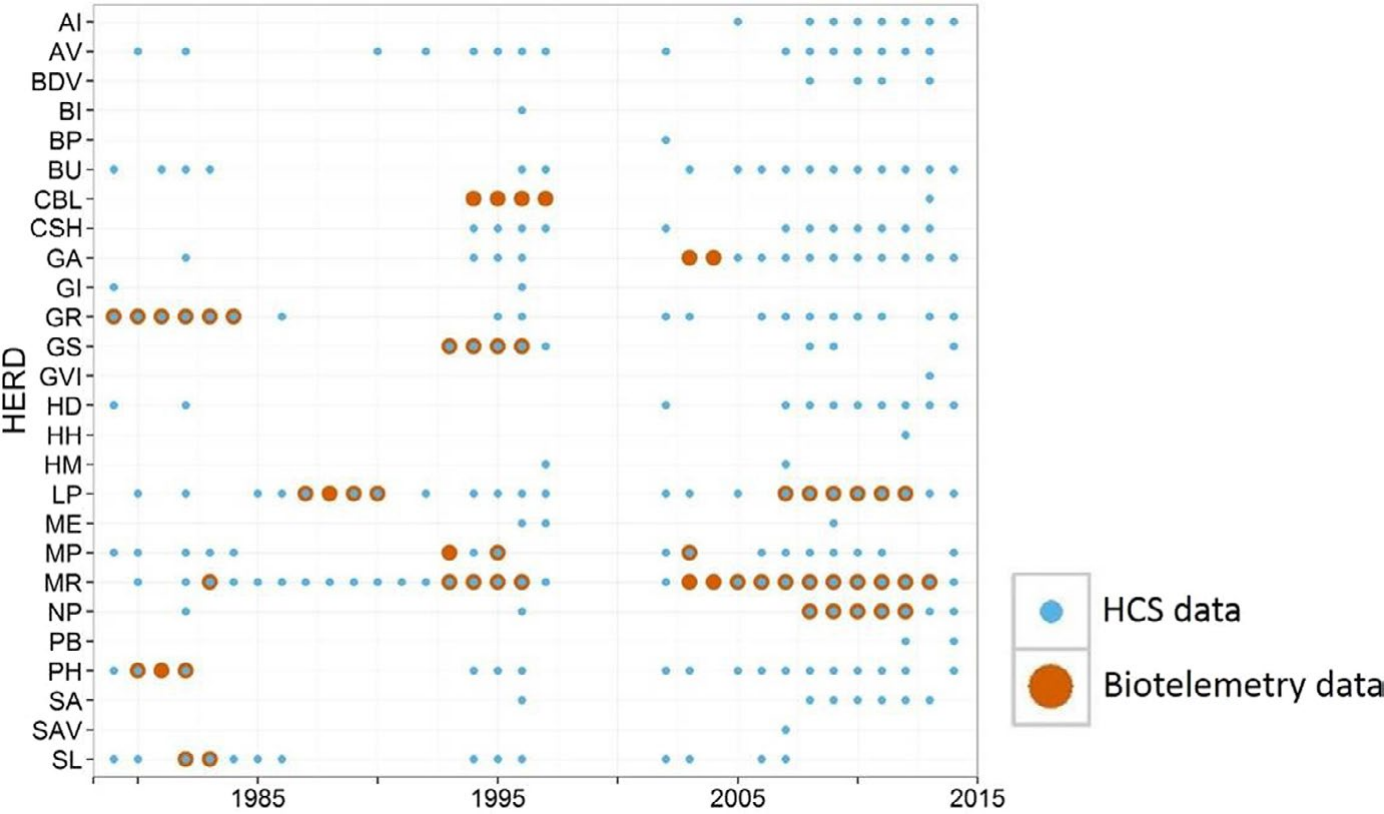
Years for which woodland caribou (*Rangifer tarandus caribou*) herd composition survey and telemetry datasets were available for different herds in Newfoundland, Canada, from 1979 to 2014. The herd abbreviations are as follows: Adies Lake (AI), Avalon (AV), Bay de Verde (BDV), Brunette Island (BI), Burin Peninsula (BP), Buchans (BU), Corner Brook Lake (CBL), Cape Shore (CSH), Gaff Topsails (GA), Grey Islands (GI), Grey River (GR), Gros Morne (GS), Glover Island (GVI), Hampden Downs (HD), Hodges Hill (HH), Humber Valley (HM), La Poile (LP), Merasheen Island (ME), Mount Peyton (MP), Middle Ridge (MR), Northern Peninsula (NP), Powerhouse Bogs (PB), Pot Hill (PH), St. Anthony (SA), Southern Avalon (SAV), and Sandy Lake (SL)

**FIGURE 6:**
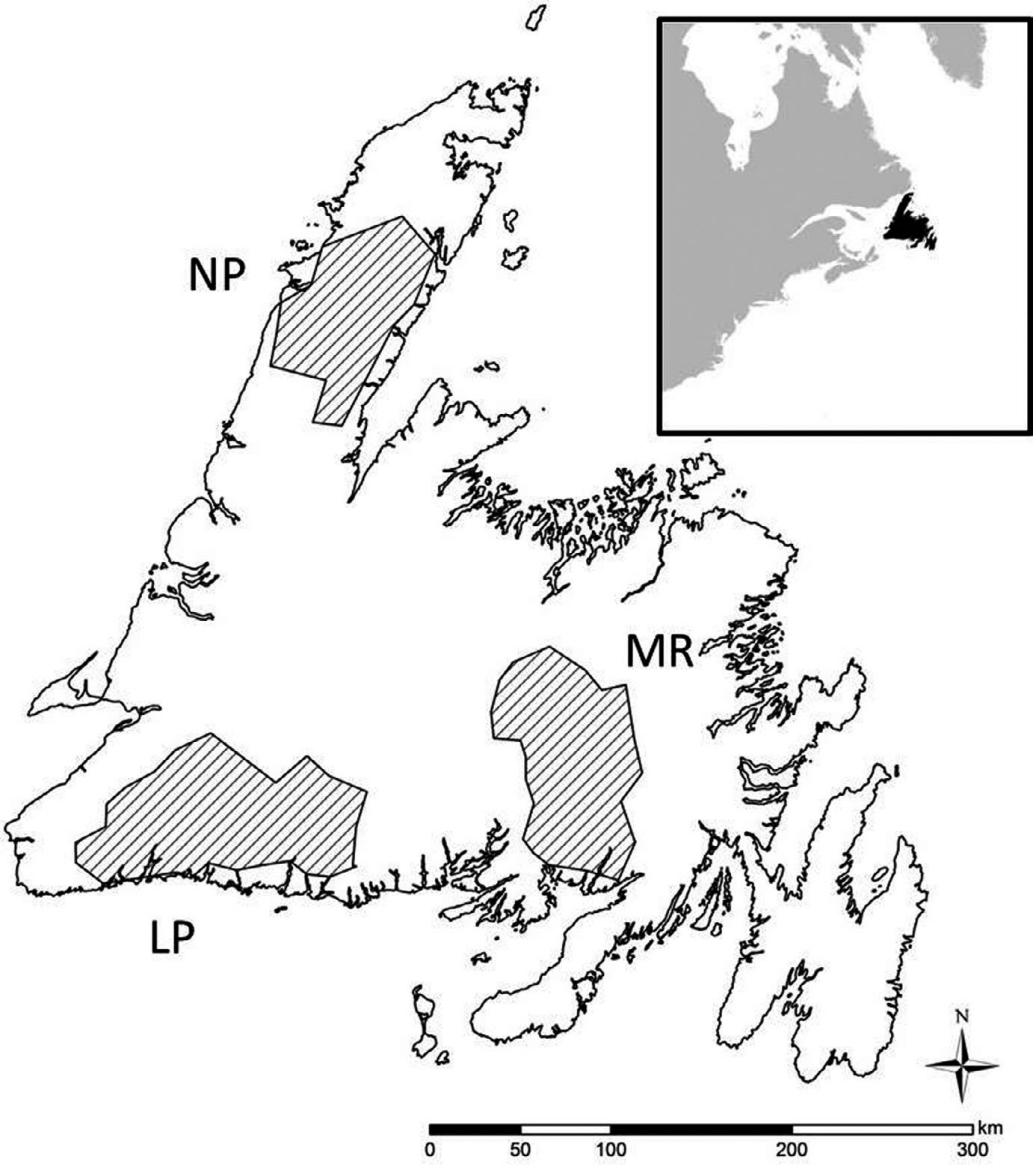
Woodland caribou (*Rangifer tarandus caribou*) herds in Newfoundland, Canada. La Poile (LP), Middle Ridge (MR), and Northern Peninsula (NP)

Woodland caribou across 26 herds were monitored by Newfoundland and Labrador government staff using either herd composition surveys or telemetry from 1979 to 2014 (Figures 5 and 6). Following Schaefer and Mahoney (2013), we categorized woodland caribou population dynamics into two distinct phases based on estimated population abundance: growth (1979–1998) and decline (2002–2014). Accounting for phases of population growth and decline in demographic studies is critical because such phases are typically density‐dependent, and as such, age structure, reproductive rate, and other demographic and ecological processes can vary (Festa‐Bianchet, Gaillard, & Côté, 2003; Festa‐Bianchet, Gaillard, & Jorgenson, 1998). In our first objective, we used data from 10 woodland caribou herds across Newfoundland that were monitored with telemetry (8 herds and 540 calves during the population growth phase and 5 herds and 635 calves during the population decline phase; Appendix A) and from 26 herds monitored with herd composition surveys (18 herds and 107 C:C estimates during the population growth phase and 24 herds and 142 C:C estimates during the population decline phase; Appendix A). For our second objective, we focused on three herds that were studied most extensively during the population decline phase: La Poile, Middle Ridge, and Northern Peninsula (17 concurrent estimates of annual herd‐wide calf survival from 448 calves and 17 estimates of calf:parous cow ratio [derived from autumn and spring herd composition surveys; see Section 2.3]; Figure 5).

### 2.2 Telemetry data

Staff from the government of Newfoundland and Labrador monitored 540 radio‐collared calves across eight herds during the population growth phase (1979–1997) and 635 radio‐collared calves across five herds during the population decline phase (2002–2014) (Figure 6 and Appendix A). Capture methods are described in Rayl et al. (2014) and Mumma et al. (2019).

#### 2.2.1 Calf collar date as an index of calf birth date

Our first objective was to determine how the date that calves were collared influenced estimates of calf survival. Woodland caribou calves were typically collared when they were only a few days old; calves were difficult to catch beyond five days old. We therefore used the number of days since an individual was collared as an index of age. We assumed that all calves were the same age when they were collared, regardless of collaring date. An estimate of calf age, based on size and calf development, was collected in the field for some calves (38%); however, these data were not estimated consistently (e.g., age, when reported, was recorded as a closed (i.e., 2–5 days) or open (i.e., >1 day) range). Thus, we could not directly assess our assumption that calves were collared within the first few days of life. Instead, we assessed our assumption indirectly by examining the relationship between calf weight and collaring date using available calf weight data for a subset of our sample from five herds during the population decline phase (*n* = 625). We did not detect a relationship between calf weight and calf collaring date across all five herds (Appendix B), suggesting that calves collared later in the season were not heavier and thus unlikely to be significantly older than calves born earlier in the season, supporting our assumption that calves were collared within the first few days of life. Other studies have linked caribou birth timing to birth weight and found that individuals born earlier weighed more than individuals more later (Adams & Dale, 1998; Côté & Festa‐Bianchet, 2001). When we examined this relationship in our data for individual herds, we did find a negative relationship between calf collar date and calf weight in the Northern Peninsula herd (Appendix B). Furthermore, when looking at animals collared on the same date in the same herd, most animals were within 2 kg of each other (IQR < 2 kg); however, the weight difference between some outliers was as high as 5 kg (Appendix B). Together, these data suggest that most animals were collared within the first few days of life, but some individuals might have been collared at ages > 5 days. To minimize this effect, we removed obvious violations to this assumption from our analyses: calves that were collared after the calving season as older individuals or calves that weighed more than 12 kg when collared (cutoff value based on conversations with staff from the Government of Newfoundland and Labrador).

#### 2.2.2 Delineating the woodland caribou calving season

Woodland caribou calves tend to be born within the same general time frame in late spring and early summer (May–June) every year, referred to as the calving season. In Newfoundland, Canada, conventional wisdom is that the calving season begins roughly in late May and concludes by mid‐June, and the start of the calving season may vary by a few days among the different herds on the island; this could be due to variation in climate and land cover (Post, Boving, Pedersen, & MacArthur, 2003). There have not been any recent studies on the timing of woodland caribou calving in Newfoundland. Bergerud (1975), however, found that in the late 1950s and early 1960s, the calving season in Newfoundland began on 24 May. Recent work by DeMars, Auger‐Méthé, Schlägel, and Boutin (2013) has shown that ungulate parturition dates can be estimated from telemetry data on adult females. However, we did not use this method across our dataset for two reasons: (a) Adult telemetry data were not available for all of the herds and years that we had calf mortality data, and (b) recent work by Bonar, Ellington, Lewis, and Vander Wal (2018) has shown that estimating migratory woodland caribou parturition dates from telemetry is not as reliable as estimating parturition for sedentary woodland caribou.

To assess the potential influence of collaring date on calf mortality risk, we defined the calving season for each herd separately using the calf collaring dates in the telemetry dataset (1979–2013). We defined the start and end of the calving season as the earliest and latest day of the year that a calf was collared for each herd across all years. We excluded herds with low sample size (*n* < 50). Using these criteria, the start date of the calving season varied between 25 May and 29 May, depending on the herd. The average start of the calving season was 27 May, and we used this date for herds that had low sample size. The end date of the calving season ranged from 8 June to 18 June for the herds for which we had large sample sizes (*n* > 50). Interestingly, the herd with the earliest collaring date, Grey River (25 May), also had the latest collaring date (18 June). The collaring dates for Grey River suggest a calving season of 25 days, while the collaring dates of the other herds (with large sample sizes, *n* > 50) suggest a calving season that ranged between 10 and 22 days. Indeed, Bergerud (1975) found that woodland caribou calving seasons in Newfoundland lasted around 25 days, although most of calving (90%) occurred during the first 12 days. Among the collared calves in our dataset, 92% were collared during the first 12 days of the calving season. Given that calves are much more likely to be born and thus collared in the first days of the calving season, we were less likely to collar a calf on the last day of the calving season. To avoid underestimating the length of the calving season, we assumed that the calving season was 25 days for each herd, which was the maximum estimated length of the calving season from any herd and in line with estimates by Bergerud (1975) (Appendix C).

#### 2.2.3 Monitoring collared calves

The monitoring of collared calves by Newfoundland government staff declined in intensity within a year: Calves were monitored every 1–3 days in the first few months postcalving (when calves were most vulnerable) and monitoring declined to once per month through the autumn and winter. We censored all animals after 1 October, when monitoring became less frequent.

### 2.3 Herd composition survey data

The herd composition surveys had more coverage, both chronologically and across herds, than the telemetry datasets. In total, 249 herd composition surveys were conducted by Newfoundland government staff for 26 woodland caribou herds across Newfoundland from 1979 to 2014, except for the years 1998–2001. Individual herds had varying degrees of data coverage during the population growth and decline phases (Figure 5 and Appendix A). Herd composition survey methods varied both temporally and spatially from 1979 to 2014, and we did not necessarily know the exact methodology used for a specific herd‐year. We summarize below a broad picture of the methods used. Between the months of September and December, observers (typically two plus the pilot) flew rotary‐wing (and perhaps in earlier years, fixed‐wing) aircraft over an area believed to be where herds were currently located. Occasionally, telemetry data were used to estimate where a woodland caribou herd was located, and in other years, the historic position of the herd was used. Herd composition surveys were typically conducted over one day, but occasionally, surveys were conducted over multiple days if weather was poor or woodland caribou were difficult to locate. For larger herds, the herd composition survey was a sampling effort, but for smaller herds, total counts were occasionally conducted. The crew counted the number of woodland caribou within basic demographic groups: adult male, adult female, and calf, and if needed, the pilot would use the aircraft to separate large groups into more manageable subgroups for counting.

Prior to 2009, the crew attempted to maximize sample size and minimize flight distance by applying a haphazard sampling design—this was practiced due to budget, time, and logistical constraints. As such, the crew focused on larger groups of woodland caribou on the landscape and in open areas. The underlying assumption (which is not necessarily supported) was that demographic make‐up of large groups in open landscapes was representative of the demographic make‐up of the entire herd. More recently, there has been a concerted effort to recognize and attempt to minimize some of the biases inherent in the haphazard sampling effort, with the use of line transect sampling that is representative of landscape type and group size (Saunders & Strickland, 2009).

As C:C represented the joint contribution of fecundity and calf survival, herd composition surveys were also conducted during the months of May and June to estimate the proportion of parous cows (an estimate of fecundity). Cows with calves close by or with visible signs of pregnancy were considered parous. With both C:C and proportion of parous cows, one can estimate the calf:parous cow ratio, which is analogous to a herd‐wide estimate of calf survival. It should be noted that cow survival rate between the two survey periods could also influence the estimate of herd‐wide calf survival; however, natural adult mortality during this time period was rare (Mahoney & Weir, 2010; Weir et al., 2014). Lewis and Mahoney (2014) monitored survival of 424 adult caribou in Newfoundland from 2004 to 2011 and only 10 mortalities occurred due to hunting (approximately 2%). Furthermore, most hunted caribou in Newfoundland are male due to focus on trophy hunts (Weir et al., 2014), so it is possible that hunting mortality of adult females was even lower than 2%. For our analysis, we assumed no cow mortality occurred between survey periods. We estimated 95% confidence intervals for herd composition survey ratios following Czaplewski, Crowe, and McDonald (1983).

#### 2.3.1 Estimating missing data with multiple imputation

There were two different types of missing data in herd composition surveys: missing survey date (5% of surveys) or the presence of unknown adults in the count data (26% of surveys; median number of unknown adults in these surveys was 3, range was 1–23). In both cases, we used multiple imputation to estimate these missing data (Appendix D). We averaged, across all the imputed datasets, model weight (*w_i_*), adjusted *R* ^2^, *p*‐value, and other parameters reported from analyses of C:C; these values therefore have an associated standard error (*SE*). We averaged results across imputed datasets after we calculated model selection metrics (AICc, ΔAICc, *w_i_*); thus, we do not report AICc and ΔAICc, as averages of these values could have been influenced by shifting baselines (lowest AICc values and model with lowest AICc value). Model coefficients would normally have an associated *SE*, so we always reported SE with these estimates.

### 2.4 Modeling method

For the herd composition survey dataset, we used linear regression models to examine how different factors might influence C:C with the lme4 package (Bates, Mächler, Bolker, & Walker, 2014) in R. We generated eight models of C:C based on the fixed effects of survey date, herd size, and year. In each model, we included herd as a random effect on the intercept. To avoid confounding the random effect of herd with fixed effects, when a herd had less than three surveys during a population phase, we collapsed it into an “other herd” category. For the telemetry dataset, we used Cox proportional hazards models to estimate mortality risk and the factors that influence mortality risk. We generated ten models of woodland caribou calf mortality risk, focused mainly on calf collar date (an index of calf birth date) and year (as both a linear fixed effect and a random effect). We ran additional models that included herd as a random effect on the intercept. We conducted all Cox proportional hazard analyses with the package survival (Therneau, 2015b) and coxme (Therneau, 2015a) in R (R Core Team, 2018).

For both datasets, we also included models with year and herd, as these factors could index temporal or spatial changes in mortality risk and C:C. Individual herds often vary in population demographics and persist in areas with different climates and land cover; thus, it is possible that the response of calf mortality risk and the C:C to different factors might vary among herds. For both datasets, we conducted model selection with AICc (Burnham & Anderson, 2002) using the package AICcmodavg (Mazerolle, 2016) in R. We considered models with AICc model weights (*w_i_*) ≥ 0.50 as a top model, and when there was not a top model, we considered all models with *w_i_* ≥ 0.05 as having some support. As additional metrics for model support and fit, we calculated concordance values (c) for fixed‐effects models of calf mortality risk, likelihood‐ratio tests (LRT) for all models of calf mortality risk, and conditional *R* ^2^ for our models of C:C.

#### 2.4.1 Modified mortality risk and calf:cow ratios

For our objective 2, we used data from three herds to compare modified estimates of herd‐wide calf survival derived from telemetry and herd composition survey data: La Poile (2007–2012, excluding 2008), Middle Ridge (2005–2012), and Northern Peninsula (2008–2012, excluding 2011; Appendix A). Comparing estimates from both telemetry and herd composition surveys was a multistep process (Figure 4). First, we generated modified estimates of mortality risk and C:C based on factors identified in the first objective (using hazard ratios for calf mortality risk and β coefficients for C:C from the population decline phase; see results). Then, we converted the modified estimates of calf mortality risk (measured at the individual level) obtained from the telemetry dataset and C:C obtained from the herd composition survey dataset to comparable estimates of herd‐wide calf survival. We again used multiple imputation to estimate the demographic identity of unknown adults in herd composition survey data (see Section 2.3.1 and Appendix D).

To convert the telemetry data to an estimate of herd‐wide calf survival, we generated an estimate of calf mortality risk within each herd‐year to match the date that the corresponding autumn herd composition survey was conducted (i.e., we censored all calves on the day of the corresponding herd composition survey). In herd‐years where multiple herd composition surveys occurred, we used the survey conducted later in the year. We then calculated the cumulative mortality risk and converted this value to calf survival probability. Cumulative mortality risk (and calf survival probability) on a given day would be different for calves born on different days because these calves would be different ages. Thus, each different aged cohort of calves would have a different survival probability estimate. We estimated the proportion of calves born on each day of the calving season across all herds and years and then modified this estimate to a Gaussian mixture curve in R. We then generated herd‐wide calf survival estimates by averaging the different age survival probabilities weighted by the estimated proportion of different ages in a herd.

To convert the herd composition survey data into an estimate of herd‐wide calf survival, within each herd‐year, we used the proportion of parous cows (obtained for the corresponding spring herd composition survey conducted during May or June; Appendix A) to adjust our modified C:C to the calf:parous cow ratio. When an estimate of the proportion of parous cows was not available, we did not estimate the calf:parous cow ratio. We equate the calf:parous cow ratio to herd‐wide calf survival because it is an estimate of the number of calves that survive from the first spring survey, which, weather permitting, took place just before the calving peak, until the autumn survey.

#### 2.4.2 Estimating trends in herd‐wide calf survival

We plotted the two estimates of herd‐wide calf survival with 95% confidence intervals for each herd during the population decline phase. Finally, we conducted a weighted regression of herd‐wide calf survival estimates within each herd using the inverse variance to estimate trends in herd‐wide calf survival. We did this for both unmodified estimates and modified estimates. We plotted an 85% confidence interval around these estimated trend lines; the choice of an 85% confidence interval allowed for easier interpretation than the large 95% confidence intervals.

## 3 RESULTS

### 3.1 Biases in calf mortality risk estimated with telemetry data

The date that a calf was collared was an important predictor of calf mortality risk during both the population growth (*n* = 540 calves) and decline phases (*n* = 635 calves), as it was present in all the models with the some support (*w_i_* > 0.05; Table 1). During the population growth phase, there was not a sole top model (*w_i_* > 0.50); rather, there were five models with some support (*w_i_* > 0.05). In addition to collar date (which was present in all 5 of these models), these models contained an effect of linear year (2 of 5 models), random effect of year (1 of 5 models), and random effect of herd (2 of 5 models). Interestingly, the hazard ratios for collar date and linear year (when present) were very similar across all five models (Appendix E). Given the similarity between the coefficients in all the supported models, we focused our interpretation on the two models with the most support (collar date and linear year + collar date). These two models not only had the highest *w_i_*, they also had concordance index values (c) > 0.50, and the LRT values indicated these models provided significantly more explanatory power than the null model (Table 1; Appendix E). During the population decline phase, we found a sole top model, linear year + collar date (*w_i_* = 0.55; c = 0.57; LRT = 30.61), and focused our interpretation on this model (Table 1; Appendix E). We found a consistently significant, positive relationship between woodland caribou calf mortality risk and the collaring date of the calf during both the population growth and decline phases (Table 2). For each day later in the calving season that a calf was born, calf mortality risk increased by 6% (hazard ratio = 1.06, 95% CI = 1.02 to 1.09) during the population growth phase and by 6% (hazard ratio = 1.06, 95% CI = 1.02 to 1.10) during the population decline phase (Table 2). Conversely, we detected a significant, negative effect of year on calf mortality risk only during the population decline phase, whereby woodland caribou calf mortality risk decreased by 6% per year (hazard ratio = 0.94, 95% CI = 0.90 to 0.99; Table 2).

**TABLE 1:** Cox proportional hazards models of woodland caribou (*Rangifer tarandus caribou*) calf mortality risk during the population growth (*n* = 540 calves from 8 herds from 1979 to 1997) and decline phases (*n* = 635 calves from 5 herds from 2003 to 2013) in Newfoundland, Canada

Population phase	Model	*k*	AICc	ΔAICc	*w_i_*
Growth	Collar date	1	1,528.23	0.00	0.37
	Linear year + collar date	2	1,529.02	0.79	0.25
	Collar date + herd (random)	2	1,530.25	2.01	0.14
	Collar date + year (random)	2	1,530.25	2.02	0.13
	Linear year + collar date + herd (random)	3	1,531.05	2.81	0.09
	Linear year	1	1,536.34	8.11	0.01
	Null	0	1,536.68	8.45	0.01
	Herd (random)	1	1,537.60	9.36	<0.01
	Linear year + herd (random)	2	1,538.36	10.12	<0.01
	Year (random)	1	1,538.66	10.42	<0.01
Decline	Linear year + collar date	2	4,012.72	0.00	0.55
	Linear year + collar date + herd (random)	3	4,014.70	1.97	0.21
	Collar date + year (random)	2	4,015.89	3.16	0.11
	Collar date	1	4,017.27	4.54	0.06
	Linear year	1	4,018.31	5.59	0.03
	Collar date + herd (random)	2	4,018.99	6.27	0.02
	Linear year + herd (random)	2	4,020.05	7.32	0.01
	Year (random)	1	4,025.18	12.46	<0.01
	Herd (random)	1	4,037.94	25.21	<0.01
	Null	0	4,039.32	26.59	<0.01

**TABLE 2:** Hazard ratios (95% CI) from selected models of mortality risk across woodland caribou (*Rangifer tarandus caribou*) population growth (*n* = 540 calves from 8 herds from 1979 to 1997) and decline phases (*n* = 635 calves from 5 herds from 2003 to 2013) in Newfoundland, Canada

Population phase	Model	*w_i_*	Linear year (yearly)		Collar date (daily)	
			Hazard ratios (95% CI)	*p*	Hazard ratios (95% CI)	*p*
Growth	Collar date	0.37			1.06 (1.02–1.09)	<.01
	Linear year + collar date	0.25	1.02 (0.99–1.05)	.27	1.06 (1.02–1.09)	<.01
Decline	Linear year + collar date	0.55	0.94 (0.90–0.99)	.01	1.06 (1.02–1.10)	.01

### 3.2 Biases in calf:cow ratios estimated with survey data

There was a single top model (linear year + survey date + herd (random); *w_i_* = 0.59, *R* ^2^ = 0.41) for C:C during the population growth phase (*n* = 107 estimates of C:C); therefore, we focused our interpretation on only this model (Table 3; Appendix E). During the population growth phase, we detected a significant, positive relationship between C:C and survey date, such that for every 30 days later in the year that a survey was conducted, the C:C was 0.10 higher (95% CI = 0.05 to 0.14; Table 4). We also detected a significant, negative relationship between C:C and year, such that during the population growth phase, the C:C decreased by 0.006 each year (95% CI = −0.010 to −0.002), equivalent to a 0.12 decline in the C:C over the entire population growth phase (Table 4). Conversely, during the population decline phase (*n* = 142 estimates of C:C) there was not a sole top model (*w_i_* > 0.50), but all eight models had some support (*w_i_* > 0.05; Table 3). The model with the most support was herd (random) (*w_i_* = 0.20; *R* ^2^ = 0.30). Furthermore, in all other models the effects of survey date, linear year, and herd size were nonsignificant (Table 4, Appendix E).

**TABLE 3:** Average AICc model weights (*w_i_*)^a^ and adjusted conditional R^2^ values^a^ for models of calf:cow ratio across the growth (*n* = 107 estimates of calf:cow ratio from 18 herds from 1979 to 1997) and declines phases (*n* = 142 estimates of calf:cow ratio from 24 herds from 2002 to 2014) of woodland caribou (*Rangifer tarandus caribou*) population dynamics in Newfoundland, Canada

Model	*k*	Growth		Decline	
		*w_i_*	Adj. *R* ^2^	*w_i_*	Adj. *R* ^2^
Herd (random)	1	<0.01	0.09	0.20	0.31
Linear year + herd (random)	2	<0.01	0.30	0.19	0.33
Survey date + herd (random)	2	0.02	0.24	0.14	0.31
Herd size + herd (random)	2	<0.01	0.15	0.08	0.30
Linear year + survey date + herd (random)	3	0.59	0.41	0.09	0.33
Linear year + herd size + herd (random)	3	<0.01	0.30	0.13	0.32
Survey date + herd size + herd (random)	3	0.05	0.29	0.07	0.30
Linear year + survey date + herd size + herd (random)	4	0.34	0.42	0.09	0.31

^a^ These are average values as a result of the multiple imputation process. As such, these values have a measure of variance (*SE*). We excluded *SE* ≤ 0.01 for brevity.

**TABLE 4:** Coefficients (β) (95% CI) of predictors from selected top or competing models of calf:cow ratio across the growth (*n* = 107 estimates of calf:cow ratio from 18 herds from 1979 to 1997) and declines phases (*n* = 142 estimates of calf:cow ratio from 24 herds from 2002 to 2014) of woodland caribou (*Rangifer tarandus caribou*) in Newfoundland, Canada

Population phase	Model	*w_i_*	Linear year (1 year)^a^	Survey date (30 days)^a^
Growth	Linear year + survey date + herd (random)	0.59	−0.006 (−0.010 to −0.003)	0.097 (0.054 to 0.139)
Decline	Herd (random)	0.20		
	Linear year + herd (random)	0.19	−0.003 (−0.007 to 0.001)	
	Survey date + herd (random)	0.14		0.015 (−0.010 to 0.040)

^a^ These are average values as a result of the multiple imputation process. As such, these values have a measure of variance (*SE*). We excluded *SE* ≤ 0.01 for brevity.

### 3.3 Reconciling herd‐wide calf survival estimates from two types of data

We modified our estimate of calf mortality risk by the coefficient identified in the top model of calf mortality risk during the population decline phase (Table 2). We also modified our estimates of the C:C by the coefficient for survey date derived from the model survey date + herd (random) during the population decline phase. The effect of survey date from this model during the population decline phase was nonsignificant, but we still used it to modify our estimates of C:C for two reasons. First, survey date had a significant positive effect on C:C during the population growth phase, and while the trend was nonsignificant and weaker, it was positive during the population decline phase, suggesting a potential link. Second, not only did we modify our estimates by the coefficient, but also by the bounds of the 95% confidence interval. Thus, we were not only accounting for the potential effect but also the uncertainty around this potential effect.

**FIGURE 7:**
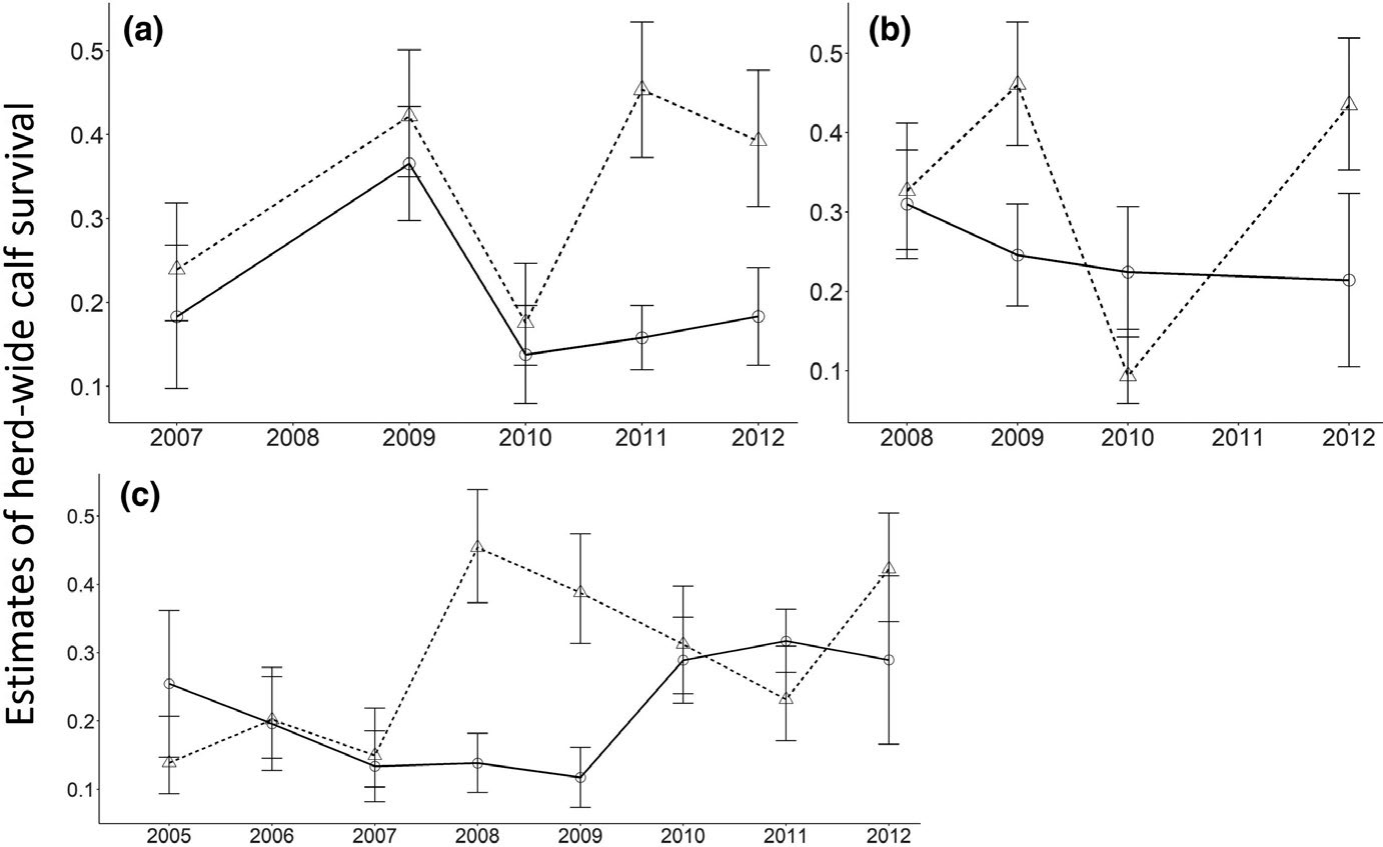
Estimates of herd‐wide woodland caribou (*Rangifer tarandus caribou*) calf survival (95% confidence intervals). Data were derived from telemetry (triangle and dashed line) and herd composition survey (circle and solid line) datasets for herds in (a) La Poile, (b) Northern Peninsula, and (c) Middle Ridge during the population decline (2003–2014) in Newfoundland, Canada. Estimates were modified to take into account the influence of collaring date (telemetry) and survey date (herd composition surveys)

**FIGURE 8:**
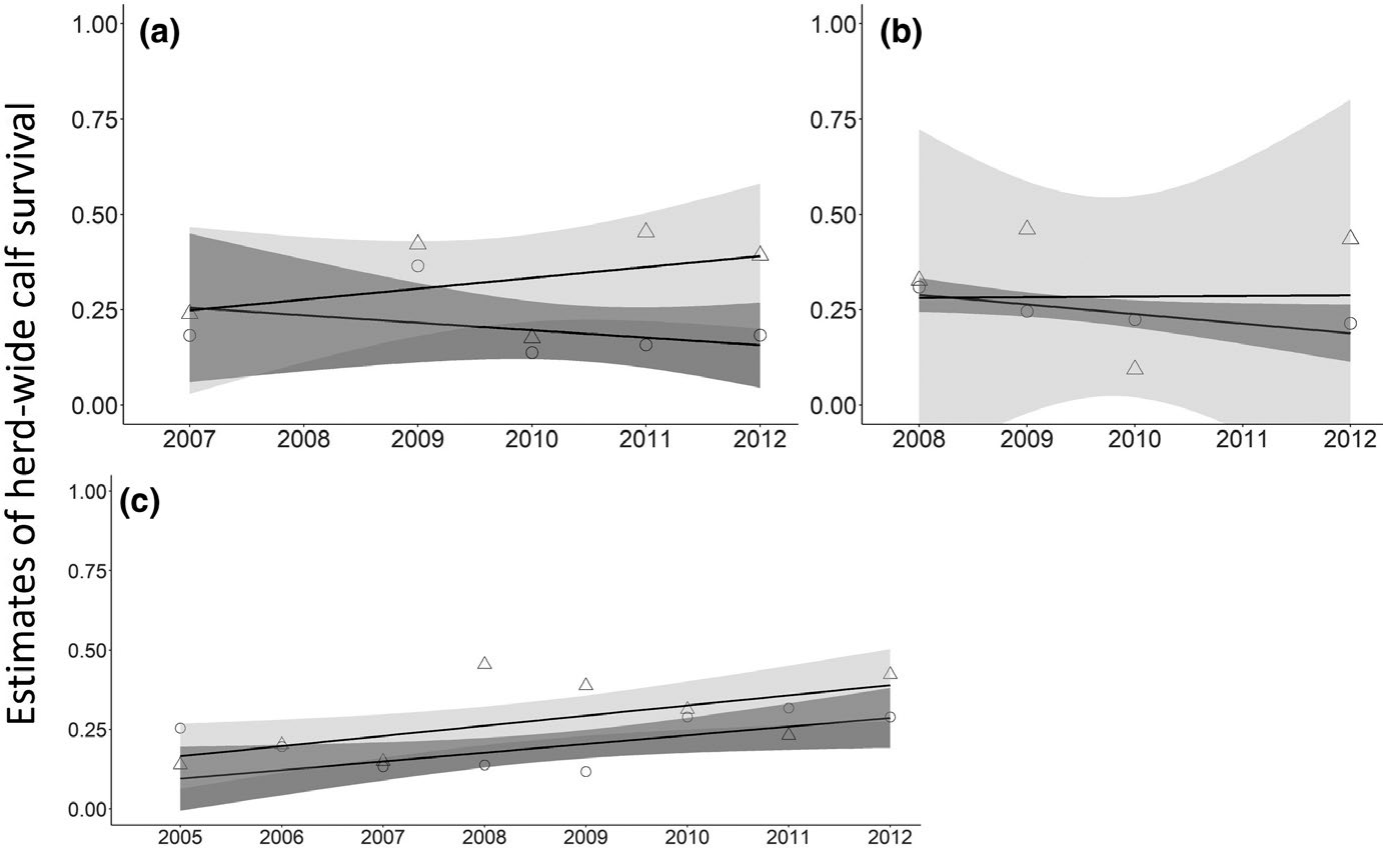
Weighted trend line (using the inverse sample variance) and 85% confidence intervals (for clarity) of estimates of herd‐wide woodland caribou (*Rangifer tarandus caribou*) calf survival. Data were derived from telemetry (triangle and light gray ribbon) and herd composition survey (circle and dark gray ribbon) datasets for herds in (a) La Poile, (b) Northern Peninsula, and (c) Middle Ridge during the population decline (2003–2014) in Newfoundland, Canada. Estimates were modified to account for the influence of collaring date (telemetry) and survey date (herd composition surveys)

When we applied the modifications to the concurrent estimates of herd‐wide calf survival during the population decline phase, we found that the 95% confidence intervals for 11 of 17 estimates overlapped (Figure 7), which is an increase from the unmodified estimates of herd‐wide calf survival, where 9 of 11 estimates had overlapping 95% confidence intervals (Figure 3). When the estimates did not overlap, estimates of herd‐wide calf survival derived from telemetry were higher than estimates from herd composition surveys (Figure 7). Nonoverlapping estimates occurred during 2008 in Middle Ridge, 2009 in Northern Peninsula and Middle Ridge, 2011 in La Poile, and 2012 in La Poile and Northern Peninsula (Figure 7). For the Middle Ridge herd, trends in herd‐wide calf survival over time derived from the modified estimates strongly agreed between the two datasets, suggesting an increase in herd‐wide calf survival over time (Figure 8c). This was an improvement over trends estimated from unmodified herd‐wide calf survival estimates in Middle Ridge, which disagreed—estimates for unmodified herd composition surveys suggested no change in herd‐wide calf survival, while estimates from unmodified telemetry suggested increasing herd‐wide calf survival. For the Northern Peninsula herd, trends in herd‐wide calf survival were not completely rectified (unmodified herd composition surveys suggested a decrease, whereas unmodified telemetry suggested an increase; Figure 3b), but agreement was improved (modified herd composition surveys suggested no change, whereas modified telemetry suggested an increase; Figure 8b). For the La Poile herd, modified estimates from telemetry data suggested that herd‐wide calf survival was increasing, whereas modified estimates from herd composition survey data suggested herd‐wide calf survival was decreasing (Figure 8a). Therefore, for the La Poile herd, our modifications did not improve estimated trends (Figure 3a). It is possible that the reduced performance of modifications in the Northern Peninsula and La Poile herds was the result of fewer available yearly estimates (*n* = 5 for La Poile and *n* = 4 for Northern Peninsula versus *n* = 8 for Middle Ridge; Figure 7).

## 4 DISCUSSION

The two most common methods for quantifying herd‐wide calf survival are herd composition surveys and telemetry. Here, we capitalized on a large, long‐term woodland caribou population monitoring dataset to compare these two methods and explore potential biases that could represent violations of equal detectability (herd composition surveys) and representative sampling (telemetry). The genesis of our study was the mismatch in calf survival estimates generated from telemetry and herd composition survey datasets. These differences cast doubt on our understanding of the state of the herd and hinder conservation and management decisions. We found that calves born later in the calving season had higher mortality risk, and this effect was consistent across both the population growth and decline phases. We also found that herd composition surveys conducted later in the year had higher estimates of C:C—we detected a significant effect during the population growth phase and a smaller, nonsignificant effect during the population decline phase. Using these relationships, we attempted to account for nonrepresentative sampling of calves (relative to calf birth date) and survey date (which might represent a violation of equal detectability). We were able to reconcile some estimates of herd‐wide calf survival by compensating for these biases in the datasets (11 of 17). In the Middle Ridge herd, the herd for which we had the most data, we were also able to reconcile the trends: Between 2005 and 2012, herd‐wide calf survival was low but showed an increasing trend.

### 4.1 Monitoring population demographics with telemetry data

While our case study was species‐specific, our findings indicate that nonrepresentative sampling of juveniles in telemetry datasets in general could introduce bias in estimates of population demographics and should be addressed in future research and monitoring efforts. For woodland caribou in Newfoundland, calves born later in the calving season had a higher mortality risk, and when herds were not representatively sampled relative to calf birth date, bias was introduced. While the link between juvenile survival and birth date has been established in many taxa (e.g., Dzus & Clark, 1998; Plard et al., 2015), other factors have also been linked to juvenile survival, including maternal condition (Taillon, Brodeur, Festa‐Bianchet, & Côté, 2012) and birth mass (Couturier, Côté, Otto, Weladji, & Huot, 2009), and these factors are often not mutually exclusive (Feder, Martin, Festa‐Bianchet, Bérubé, & Jorgenson, 2008). Thus, in our system and in other systems, bias in estimates of juvenile survival could arise from different ecological factors. Furthermore, ecological factors such as birth date can differentially influence estimates of juvenile survival across nested temporal scales. For example, in many species, survival is greatly increased when births are synchronized, and calf survival tends to be lower for those individuals born outside of the birthing season (Rutberg, 1987). Within the birthing season, fawn survival might be higher for those individuals born close to the peak birth date (Jarnemo, Liberg, Lockowandt, Olsson, & Wahlström, 2004) or higher for those individuals born farther from the peak birth date (Aanes & Andersen, 1996). Finally, diel timing of birth could also influence survival (Patterson, Mills, Middel, Benson, & Obbard, 2016). These nuances further the argument that monitoring efforts should carefully consider the ecology of the species and the ecosystem in which they occur and plan sampling methods accordingly.

For woodland caribou in Newfoundland, our results indicated that the effect of calf birth date on mortality risk was equivalent during the population growth and decline phases. The increased mortality risk for calves born later in the season could be driven by access to resources, vulnerability to predators, or both, and therefore, we might expect the magnitude of this risk to vary depending on climatic conditions or fluctuations in predator populations. Among some ungulate species, it has been suggested that when predation rates are high, birth date has no influence on fawn survival (Fairbanks, 1993). However, in Newfoundland we found evidence of birth date influencing calf survival, despite predation being the dominant source of mortality for collared woodland caribou calves (Mahoney et al., 2016). Indeed, calves were more susceptible to predation during the population decline phase than during the population growth phase (Mahoney et al., 2016), and this was driven by the relationship between maternal body condition, susceptibility to climatic events, and predation risk (Bastille‐Rousseau et al., 2016).

### 4.2 Monitoring population demographics with herd composition surveys

We also found some evidence that herd composition survey data could be biased by survey date for woodland caribou in Newfoundland. We found evidence suggesting that timing of the herd composition survey can influence the C:C, whereby surveys conducted later in the autumn tended to have a higher C:C. One potential explanation for this counterintuitive result is that different demographic groups might aggregate at different times. Prior to calving, herds break apart as cows move away from other individuals to calve. Postcalving, cows and calves gradually rejoin other individuals, gradually resulting in the reconstitution of the herd for the winter. Perhaps cows that lose calves or nonreproductive cows rejoin the aggregated herd earlier. This highlights the importance of careful attention to social behavior in social ungulates. Future research with collared individuals could explore herd aggregation patterns relative to different demographic groups. Indeed, in western Greenland, caribou movement and aggregation patterns changed from year to year, impacting demographic estimates from herd composition surveys (Poole, Cuyler, & Nymand, 2013).

Beyond our case study, studies monitoring a variety of species using herd composition surveys could be afflicted with violations of equal detectability (e.g., walrus (*Odobenus rosmarus*; Citta et al., 2014). Further, in populations that experience changes in population abundance, the factors that influence detectability may change, which can result in misleading results and incorrect conservation decisions (e.g., the endangered Saiga antelope (*Saiga tatarica*; McConville et al., 2009). Among ungulates, accounting for unequal and imperfect detectability in herd composition surveys has been an important component of wildlife research and management for many years (Caughley, 1974). It should be noted that managers can attempt to minimize these biases, for example, by using route and timing standardization for herd composition surveys (McCullough, 1994). Such strategies are not uniformly adopted, however, and can be cost‐prohibitive in certain systems. While we were only able to explore how survey date and herd size might have influenced equal detectability among demographic groups, many other methodological factors could have played a role, including number of observers, observer experience, and effort (flight transect spacing, total distance, etc.). Furthermore, variables such as weather and underlying land cover could have had an unpredictable effect on estimates of C:C (Vander Wal, McLoughlin, & Brook, 2011). Patterson, Drake, Allen, and Parent (2014), however, found that many of these factors did not influence C:C for caribou in Ontario, Canada. Additionally, a preliminary analysis did not reveal a relationship between C:C and the number of woodland caribou groups or the average group size (Ellington, 2015).

### 4.3 Analytical considerations

With an analysis of this scale and scope, there are bound to be limitations. We assumed that collar date was an index of calf birth date, and we demonstrated that there was no relationship between calf birth weight (an index of calf age) and calf collar date. There could, however, have been violations of this assumption (Appendix B). Indeed, other studies have concluded that lighter birth weight and/or later birth date could be linked via maternal condition (Adams, 2005; Cameron, Smith, Fancy, Gerhart, & White, 1993; Festa‐Bianchet, 1988; Verme, 1989). Broadly speaking, caribou calf birth weight may have been higher in Newfoundland during the population growth phase than during the population decline phase (Mahoney & Weir, 2010), which is similar to other caribou herds (Couturier et al., 2009). If there were individuals in our dataset that were collared as older individuals (i.e., individuals born earlier in the calving season masquerading as individuals born later in the season), then we would have underestimated the effect of calf birth date on calf mortality risk.

To properly account for unrepresentative sampling bias of calves relative to calf birth date within a season, the distribution of calf birth dates within a season must be known. We generated this using the average distribution across all herds and years, but an independent estimate of this distribution would be preferable. Some potential methods of estimating the calving season include the use of vaginal implant transmitters on a sample of adult females, and some researchers have had success estimating parturition from the movement patterns of adult females (DeMars et al., 2013 but see Bonar et al., 2018). While the parturition date among some ungulates is highly repeatable (Plard et al., 2013), future work should examine whether the distribution of caribou calf birth dates within a calving season varies with climate, population density, herd, and maternal age, condition, and sociality. Indeed, date of the calving season has changed over time in the George River caribou herd in Quebec and Labrador, Canada (Couturier, Brunelle, Vandal, & St‐Martin, 1990), and recent work has shown that caribou calves in Newfoundland are occasionally born beyond the typical 25‐day calving season (Bonar, Laforge, & Vander Wal, 2017). Such variation, if not properly accounted for, would also result in biased estimates of herd‐wide calf survival.

The proportion of parous cows that we used to transform C:C into estimates of herd‐wide calf survival could themselves be biased by violations of equal detectability, as they were also derived from herd composition surveys. There are a few potential avenues for such violations to occur: (a) if parous females or very young calves are difficult to detect and get counted as barren females, resulting in an underestimate of parous females and an overestimate of herd‐wide calf survival; (b) if barren females are less likely to be on calving grounds than parous females (see Fifield, Lewis, & Gullage, 2012), resulting in an overestimate of parous females and an underestimate of herd‐wide calf survival; and (c) if other methodological variables, such as observer experience, effort, and weather, lead to unpredictable variation in estimates of the proportion of parous cows. Staff from the government of Newfoundland and Labrador were highly experienced in composition surveys and confident in their ability to distinguish reproductive status of cows and identify young calves in the primarily open landscapes of the calving grounds. Therefore, we think it is unlikely that parous females were underestimated or that unpredictable variation occurred due to observer experience or effort (especially during the population decline phase). Estimates of the proportion of parous females were fairly constant for herd‐years in our comparison of the La Poile and Middle Ridge herds but were lower in the Northern Peninsula herd, and there was one outlier year for the La Poile herd (Appendix A and Weir et al., 2014). Future work should identify when and if different female demographic groups arrive at the calving grounds. If animals are monitored with telemetry data, then vaginal implant transmitters (Kaze, Whiting, Freeman, Bates, & Larsen, 2016) or movement characteristics might also allow managers to estimate parturition rate among cows independently from herd composition surveys (DeMars et al., 2013 but see Bonar et al., 2018). Furthermore, telemetry studies could also examine herd aggregation patterns after calving relative to different demographic groups and such studies could form an important link between the relationship between survey date and equal detectability of demographic groups.

Herd composition surveys are a traditional and relatively inexpensive way to monitor a multitude of demographic parameters but there could be hidden variability. We caution that future interpretations of herd composition survey data are cognizant of this limitation. Misclassifying individuals in composition surveys will lead to bias; when observers are uncertain about the demographic classification, they should classify these individuals as unknown. By using multiple imputation, we were able to avoid masking the effect of unknown individuals on our ratios while at the same time accounting for the variability caused by unknown individuals in herd composition survey data. We also note that herd composition surveys cannot provide the additional data, such as timing and cause of death, that telemetry can provide; without telemetry data, the mechanisms underlying population changes are difficult to understand. Further, as suggested by Citta et al. (2014), understanding and mitigating some of the biases of herd composition surveys likely requires occasional comprehensive telemetry studies. Finally, more broadly, Harris et al. (2008) found that C:C performed poorly at detecting declines in calf survival and argue that the inclusion of independent estimates of calf survival, such as from telemetry, is important when populations need to be monitored closely.

Our study would not have been possible without the long‐term monitoring datasets collected on woodland caribou herds across Newfoundland over the last 40 years. While our study found issues with both monitoring methods, we did not assess the validity of past research using these datasets and we caution against dismissing past studies and conclusions using these datasets without a thorough reassessment. We do not, however, support one monitoring method over the other. Instead, we recommend improvements to both monitoring methods. Ultimately, a comprehensive multi‐approach monitoring program produces higher quality data, which could lead to more concrete management recommendations.

### 4.4 Concluding thoughts

Ecological data are inherently noisy. Numerous ecological processes can influence calf mortality risk and recruitment rate (C:C), and moreover, they can vary among populations and ecosystems, and in response to climate change (Osinga, Pen, de Haes, & Brakefield, 2012), food availability (Bowen, Ellis, Iverson, & Boness, 2003), or human interactions (Gamelon et al., 2011). Demographic factors such as birth date can have impacts beyond juvenile survival; they can impact dispersal patterns (Jansen & Jenks, 2012) and overall fitness (Green & Rothstein, 1993), and can in some instances have stabilizing effects (e.g., Schultz, 1993). Not accounting for methodological biases when using different sampling methods to estimate ecological phenomenon and population demographics could have unexpected and unknown impacts. Even if we could control for or eliminate methodological biases, there would still be, and should be, a measure of uncertainty around estimates of herd‐wide calf survival, some of which may be ecological and some merely stochastic.

## CONFLICT OF INTEREST

We declare we have no competing interests.

## AUTHOR CONTRIBUTIONS

**Edward Hance Ellington:** conceptualization (equal); formal Analysis (lead); Funding acquisition (equal); Investigation (lead); Methodology (lead); Writing‐original draft (lead); Writing‐review & editing (lead). **Keith P. Lewis:** Conceptualization (equal); Data curation (supporting); Funding acquisition (equal); Methodology (supporting); Writing‐original draft (supporting); Writing‐review & editing (supporting). **Erin L. Koen:** Writing‐original draft (supporting); Writing‐review & editing (supporting). **Eric Vander Wal:** Conceptualization (equal); Funding acquisition (equal); Supervision (supporting); Writing‐original draft (supporting); Writing‐review & editing (supporting).

## Figures and Tables

**FIGURE A1:**
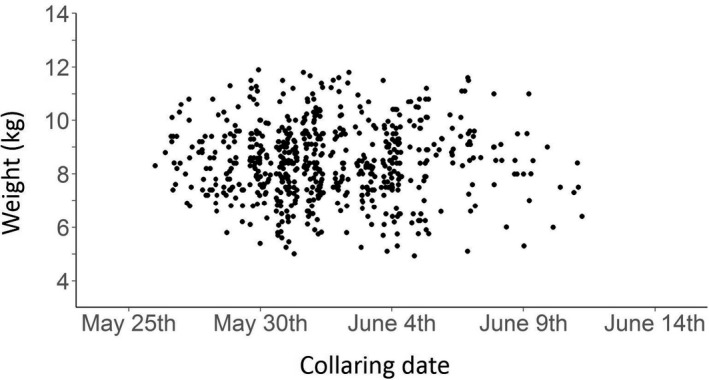
Estimates of herd‐wide woodland caribou (Rangifer tarandus caribou) calf:cow ratio (triangle and dashed line) and calf:parous cow ratio (circle and solid line) from herd composition survey datasets for herds in (a) La Poile, (b) Northern Peninsula, and (c) Middle Ridge during the population decline (2003–2014) in Newfoundland, Canada. Estimates represent unmodified data

**FIGURE B1:**
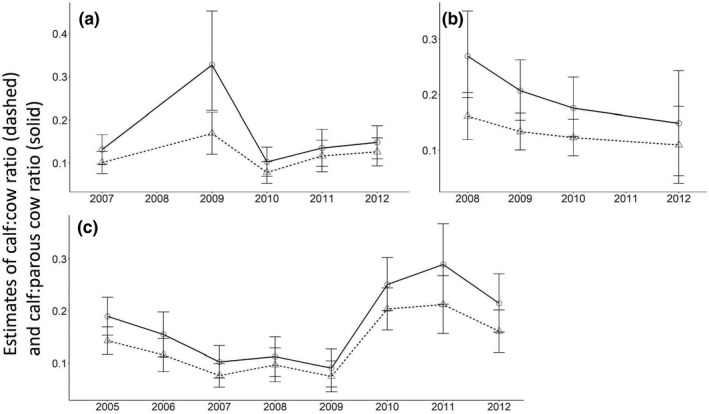
The relationship between woodland caribou calf (*Rangifer tarandus caribou*) collaring date and calf weight (kg) across five herds (Gaff Topsails, La Poile, Mount Peyton, Middle Ridge, and Northern Peninsula) in Newfoundland, Canada (*n* = 625 calves), from 2003 to 2013. This relationship was not significant (linear regression collaring date *β* = −0.001 [*SE* = 0.02]; *p*‐value = .96)

**FIGURE B2:**
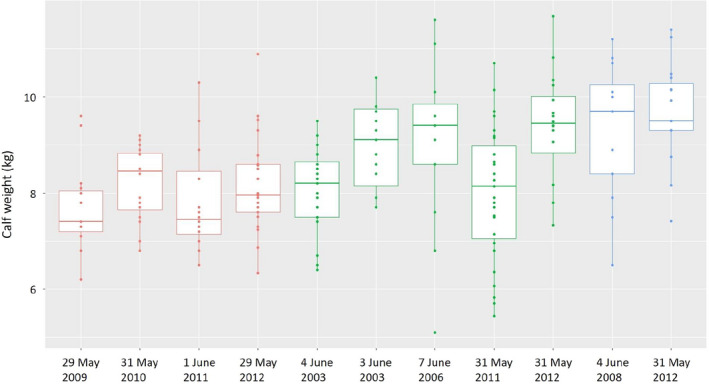
Select herd, years, and days when 15 or more woodland caribou calves (*Rangifer tarandus caribou*) were collared and weights of these individuals with box plots to show interquartile range. La Poile herd (red), Middle Ridge herd (green), Northern Peninsula herd (blue)

**FIGURE C1:**
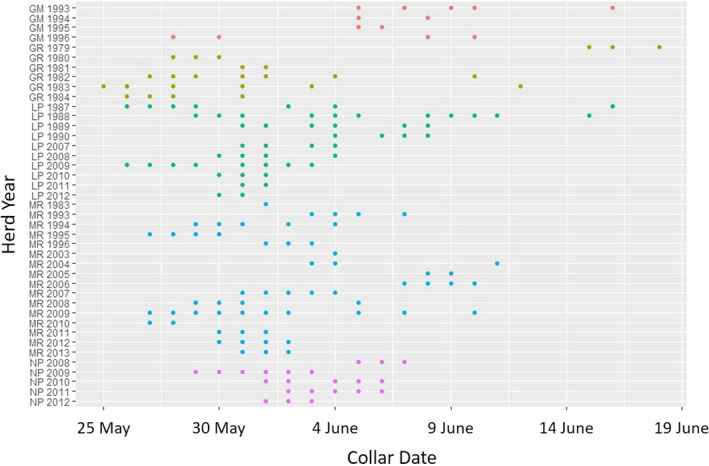
Distribution of dates that calves were collared across five woodland caribou (*Rangifer tarandus caribou*) herds with at least 50 calves collared. The herd abbreviations are as follows: Gros Morne (GM), Grey River (GR), La Poile (LP), Middle Ridge (MR), and Northern Peninsula (NP)

## Data Availability

All data used for this analysis are available on the Dryad Digital Repository at https://doi.org/10.5061/dryad.xgxd254db

## Appendix A

### DATA SUMMARIES

This appendix contains summary data on telemetry studies used to estimate woodland caribou (*Rangifer tarandus caribou*) calf mortality risk (Table A1), summary data on autumn herd composition surveys used to estimate woodland caribou calf:cow ratio (Table A2), and summary data on spring herd composition surveys used to estimate woodland caribou parous cow:cow ratio (Table A3). This appendix also includes summary data on the telemetry studies and herd composition surveys used to compare estimates and trends of herd‐wide calf survival (Table A4). This appendix also shows the difference between calf:cow ratio and calf:parous cow ratio (calculated from calf:cow ratio derived from autumn herd composition surveys and parous cow:cow ratio derived from spring herd composition surveys) for the herd‐years during the population decline phase (2002–2014) where we had both telemetry and herd composition survey data (Figure A1).

**TABLE A1:** Telemetry datasets used to estimate woodland caribou (*Rangifer tarandus caribou)* calf mortality risk during the population growth (1979–1997) and declines phases (2002–2013) in Newfoundland, Canada

Population Phase	Herd	Years	Sample size
Growth	Corner Brook Lake	1994–1997	45
	Grey River	1979–1984	174
	Gros Morne	1993–1996	69
	La Poile	1987–1990	123
	Mount Peyton	1993, 1995	10
	Middle Ridge	1983, 1993–1996	86
	Pot Hill	1980–1982	14
	Sandy Lake	1982–1983	19
Decline	Gaff Topsails	2003–2004	48
	La Poile	2007–2012	175
	Middle Ridge	2003–2013	273
	Mount Peyton	2003	9
	Northern Peninsula	2008–2012	130

**TABLE A2:** Autumn herd composition surveys used to estimate woodland caribou (*Rangifer tarandus caribou)* calf:cow ratio during the population growth (1979–1997) and declines phases (2002–2013) in Newfoundland, Canada

Population phase	Herd	Years	Sample size^a^	Notes
Growth	Avalon	1980, 1982, 1990, 1992, 1994–1997	9	Two surveys conducted in 1996
	Brunette Island	1996	1	
	Buchans	1979, 1981–1983, 1996, 1997	6	
	Cape Shore	1994–1997	4	
	Gaff Topsails	1982, 1994–1996	5	Two surveys conducted in 1982
	Grey Island	1979, 1996	2	
	Grey River	1979–1984, 1986, 1995, 1996	9	
	Gros Morne	1993–1997	5	
	Hampden Downs	1979, 1982	2	
	Humber Valley	1997	1	
	La Poile	1980, 1982, 1985–1987, 1989, 1990, 1992, 1994–1997	15	Two surveys conducted in 1987 and three surveys conducted 1992
	Merasheen Island	1996, 1997	2	
	Middle Ridge	1980, 1982–1997	19	Two surveys conducted in 1991 and 1992
	Mount Peyton	1979, 1980, 1982–1984, 1994, 1995	7	
	Northern Peninsula	1982, 1996	2	
	Pot Hill	1979, 1980, 1982, 1994–1996	7	Two surveys conducted in 1982
	St Anthony	1996	1	
	Sandy Lake	1979, 1980, 1982–1986, 1994–1996	10	
Decline	Adies Lake	2005, 2008–2014	8	
	Avalon	2002, 2007–2013	8	
	Bay de Verde	2008, 2010, 2011, 2013	4	
	Burin Peninsula	2002	1	
	Buchans	2003, 2005–2014	11	
	Corner Brook Lake	2013	1	
	Cape Shore	2002, 2007–2013	8	
	Gaff Topsails	2005–2014	10	
	Glover Island	2013	1	
	Grey River	2002, 2003, 2006–2011, 2013, 2014	12	Three surveys conducted in 2003
	Gros Morne	2008, 2009, 2014	3	
	Hampden Downs	2002, 2007–2014	9	
	Humber Valley	2007	1	
	Hodges Hills	2012	1	
	La Poile	2002, 2003, 2005, 2007–2014	11	
	Merasheen Island	2009	1	
	Middle Ridge	2002, 2005–2014	11	
	Mount Peyton	2002, 2003, 2006–2011, 2014	9	
	Northern Peninsula	2008–2014	8	Two surveys conducted in 2008
	Pot Hill	2002, 2003, 2005–2012, 2014	11	
	Powerhouse Bogs	2012, 2014	2	
	St Anthony	2008–2013	6	
	Sandy Lake	2002, 2003, 2006, 2007	4	
	Southern Avalon	2007	1	

^a^ Number of surveys conducted in the autumn for each herd and for each phase.

**TABLE A3:** Spring woodland caribou (*Rangifer tarandus caribou*) herd composition surveys that were available to estimate proportion of parous females during the population growth (1979–1997) and declines phases (2002–2013) in Newfoundland, Canada

Population phase	Herd	Years	Notes
Growth	Avalon	1992, 1996	2 surveys conducted in 1996
	Gaff Topsails	1996	
	Grey River	1979–1984, 1995, 1996	3 surveys conducted in 1982 and 2 surveys conducted in 1984
	Gros Morne	1993–1997	3 surveys conducted in 1995 and 3 surveys conducted in 1997
	La Poile	1980, 1987, 1990, 1996	
	Merasheen Island	1996	
	Middle Ridge	1996	
	Mount Peyton	1982, 1995	
	Pot Hill	1980, 1982, 1995, 1996	
	Sandy Lake	1980, 1995, 1996	
Decline	Avalon	2008, 2010	
	Buchans	2007, 2009, 2011, 2012	
	Cape Shore	2002, 2007, 2010, 2011	
	Gaff Topsails	2006−2009, 2011, 2012	
	Grey River	2002, 2003, 2006, 2007	
	Hampden Downs	2007, 2012	
	La Poile	2002, 2005, 2007, 2009–2012	
	Middle Ridge	2002, 2005–2012	
	Mount Peyton	2002, 2003, 2006–2011	
	Northern Peninsula	2008–2010, 2012	
	Pot Hill	2002, 2003, 2005–2012	2 surveys conducted in 2002
	Sandy Lake	2002, 2006	

**TABLE A4:** Woodland caribou (*Rangifer tarandus caribou*) herd composition surveys and telemetry data collected in the autumn of the same year that we used to modify and convert estimates of calf:cow ratio and calf mortality risk into herd‐wide estimates of calf survival for each herd and year during the population decline phase in Newfoundland, Canada

Herd	Year	Number of calves collared	Herd composition survey notes
La Poile	2007	31	
	2009	40	
	2010	24	
	2011	25	
	2012	25	
Middle Ridge	2005	16	
	2006	23	
	2007	25	
	2008	24	
	2009	33	
	2010	16	
	2011	31	
	2012	29	
Northern Peninsula	2008	28	2 surveys conducted
	2009	38	
	2010	16	
	2012	24	

## Appendix B

### RELATIONSHIP BETWEEN CALF WEIGHT AND COLLAR DATE

This appendix explores the relationship between the collar date (capture date and assumed index of birth date) and weight at capture of woodland caribou calves (*Rangifer tarandus caribou*) from Newfoundland. These individuals were captured as part of a long‐term monitoring effort by the government of Newfoundland and Labrador. These data were a subset of the data we used to estimate biases potentially influencing estimates of herd‐wide calf survival from telemetry data. We found no relationship between calf weight and collar date (linear regression collaring date *β* = −0.001 [*SE* = 0.02]; *p*‐value = .96; Figure B1). We also conducted a linear regression analysis on individual herds that had at least 50 calves with weight data. We found no relationship between calf weight and collar date in La Poile (*β* = −0.02 [*SE* = 0.05], *p* = .66, *n* = 174) or Middle Ridge (*β* = −0.01 [*SE* = 0.02], *p* = .81, *n* = 267). However, we did find a significant negative relationship between calf weight and collar date in the Northern Peninsula herd (*β* = −0.10 [*SE* = 0.04], *p* = .03, *n* = 127): Calf weight decreased by 0.10 kg per day. For the Northern Peninsula herd, our samples spanned a total of 10 days, so animals collared at the earliest point in the calving season sampled were 1 kg heavier than animals collared at the latest point in the calving season sampled. We further investigated individual herd‐dates where ≥ 15 calves were captured, across these herd‐dates, the interquartile range was less than 2 kg (Figure B2). However, in two herd‐year‐dates, Middle Ridge 7 June 2006 and Middle Ridge 31 May 2011, some outlier calves were more than double the weight of some other outlier calves (Figure B2).

## Appendix C

### ESTIMATING CARIBOU CALVING SEASON

This appendix explores collar date of woodland caribou calve (*Rangifer tarandus caribou*) in our telemetry dataset. We report the day of the year that the first caribou calf was collared and the last day of the year a caribou calf was collared for each herd (Table C1), and we also show distribution of these dates across each herd (Figure C1). For herds with large sample sizes (*n* > 50), we used the first calf collar date as the beginning of the calving season. For herds with small sample sizes (*n* < 50), we used the average calving season start date of 27 May. All calving seasons were 25 days long.

**TABLE C1:** Dates of first and last woodland caribou (*Rangifer tarandus caribou)* calf collared, and estimated dates of the calving season by herd in Newfoundland, Canada, from 1979 to 2013

Herd	Sample size	First calf collared	Last calf collared	Beginning of calving season	End of calving season
Grey River	174	25 May	18 June	25 May	18 June
Gros Morne	69	28 May	16 June	28 May	21 June
La Poile	298	26 May	16 June	26 May	19 June
Middle Ridge	359	27 May	11 June	27 May	20 June
Northern Peninsula	130	29 May	7 June	29 May	22 June
Gaff Topsails	48	3 June	7 June	27 May	20 June
Mount Peyton	19	27 May	4 June	27 May	20 June
Pot Hill	14	1 June	1 June	27 May	20 June
Sandy Lake	19	27 May	4 June	27 May	20 June
Corner Brook Lake	45	27 May	16 June	27 May	20 June

## Appendix D

### MULTIPLE IMPUTATION OF HERD COMPOSITION SURVEY DATA

Two types of missing or incomplete data were present in woodland caribou *(Rangifer tarandus caribou)* herd composition survey data in Newfoundland, Canada. The first type of incomplete or missing data was the date of the survey: Either the month of the survey or the day of the survey was missing. When the month of the survey was unknown, we could not be certain whether the survey was conducted during the autumn; thus, we removed these data from the analysis. When the month was known but the day of the survey was missing, we used multiple imputation to estimate the missing day. We first converted day to a proportion of day of the month (i.e., the 15th became 15/30 for September and November or 15/31 for October and December]). After transforming the proportion to a continuous value, we used multiple imputation to estimate the missing day of the month.

The second type of missing data in the herd composition survey dataset came in the form of unknown adult caribou. When observers were unable to determine the sex of an adult caribou, they recorded it as an unknown adult. Unfortunately, this can introduce bias because an unknown ratio of these “unknown adults” is adult females and thus should be included in the calf:cow ratio. To estimate the proportion of unknown adults that were female, we first calculated the proportion of adult females/adult for every dataset without unknown adults. We then transformed this proportion to a continuous value, used multiple imputation to estimate the number of missing adult females/adult, multiplied this value by the number of unknown adults, and added it to the recorded number of adult females.

To conduct the multiple imputation, we iteratively drew estimates for each of these missing values using a conditional distribution of each missing value given the observed data. This process approximates a Bayesian framework—multiple chains are run, and convergence is assessed after a specified number of iterations. We conducted both multiple imputation procedures sequentially: First, we imputed unknown adults, and then, we imputed day of the month. We generated a total of 100 imputed datasets. We conducted all the multiple imputation processes using the package mice (van Buuren & Groothuis‐Oudshoorn, 2010) in R (R Core Team, 2017). We used the R package BaBooN (Meinfelder & Schnapp, 2015) to summarize model coefficients and associated standard errors. Within the AIC framework, we followed Nakagawa and Freckleton (2011) and first generated AICc weights for each model of each imputed dataset and then summarized the model weights.

## Appendix E

### ADDITIONAL MODEL RESULTS

In this appendix, we report the full results (model selection metrics, model fit metrics, coefficients, and hazard ratios) for all models of woodland caribou calf (*Rangifer tarandus caribou*) mortality risk in Newfoundland, Canada, during the population growth phase (*n* = 540, 1979–1997; Table E1) and the population decline phase (*n* = 635, 2003–2013; Table E2). We also report the full results (model selection metrics, model fit metrics, and coefficients) for all models of woodland caribou calf:cow ratios in Newfoundland, Canada, during the population growth phase (*n* = 107, 1979–1997; Table E3) and the population decline phase (*n* = 142, 2002–2014; Table E4).

**TABLE E1:** Cox proportional hazards models and the effects of collar date and linear year on woodland caribou (*Rangifer tarandus caribou*) calf mortality risk (*n* = 540) during the population growth phase across eight herds in Newfoundland, Canada, from 1979 to 1997

Model	Model selection metrics				Measures of model fit^a^		Coefficient estimates (*SE*)		Hazard ratios (95% confidence interval)	
	*k*	AICc	ΔAICc	*w_i_*	c (SE)	LRT (*SE*, *df*)	Collar date	Linear year	Collar date (daily)	Linear year (yearly)
Collaring date of calf	1	1,528.23	0.00	0.37	0.59 (0.03)	10.45 (<0.01, *df* = 1)	1.34 (0.39)	—	1.06 (1.02 to 1.09)	—
Collaring date of calf + linear year	2	1,529.02	0.79	0.25	0.60 (0.02)	11.68 (<0.01, *df* = 2)	1.29 (0.40)	0.31 (0.28)	1.06 (1.02 to 1.09)	1.01 (0.99 to 1.05)
Collaring date of calf + caribou herd (random)	2	1,530.25	2.01	0.14	—	10.46	1.34 (0.39)	—	1.06 (1.02 to 1.09)	—
Collaring date of calf + year (random)	2	1,530.25	2.02	0.13	—	10.45	1.34 (0.39)	—	1.06 (1.02 to 1.09)	—
Collaring date of calf + linear year + caribou herd (random)	3	1,531.05	2.81	0.09	—	11.68	1.29 (0.40)	0.31 (0.28)	1.06 (1.02 to 1.09)	1.01 (0.99 to 1.05)
Linear year	1	1,536.34	8.11	0.01	0.54 (0.03)	2.35 (0.13, *df* = 1)	—	0.43 (0.28)	—	1.02 (0.99 to 1.06)
Null	0	1,536.68	8.45	0.01	—	—	—	—	—	—
Caribou herd (random)	1	1,537.60	9.36	0.00	—	1.09	—	—	—	—
Linear year + caribou herd (random)	2	1,538.36	10.12	0.00	—	2.35	—	0.43 (0.28)	—	1.02 (0.99 to 1.06)
Year (random)	1	1,538.66	10.42	0.00	—	0.03	—	—	—	—

^a^ Concordance index (c) was estimated from fixed‐effects models, and likelihood‐ratio test (LRT) comparing model with null model was estimated for fixed‐ and mixed‐effects models.

**TABLE E2:** Cox proportional hazards models and the effects of collar date and linear year on woodland caribou (*Rangifer tarandus caribou*) calf mortality risk (*n* = 635) during the population decline phase across five herds in Newfoundland, Canada, from 2003 to 2013

Model	Model selection metrics				Measures of model fit		Coefficient estimates (*SE*)		Hazard ratios (95% confidence interval)	
	*k*	AICc	ΔAICc	*w_i_*	c (*SE*)	LRT (*SE*, *df*)	Collar date	Linear year	Collar date (daily)	Linear year (yearly)
Collaring date of calf + linear year	2	4,012.72	0.00	0.55	0.57 (0.02)	30.61 (<0.01, *df* = 2)	1.40 (0.50)	−0.58 (0.22)	1.06 (1.02 to 1.10)	0.94 (0.90 to 0.99)
Collaring date of calf + linear year + caribou herd (random)	3	4,014.70	1.97	0.21	—	30.65	1.38 (0.50)	−0.57 (0.23)	1.06 (1.02 to 1.10)	0.94 (0.90 to 0.99)
Collaring date of calf + year (random)	2	4,015.89	3.16	0.11	—	27.45	1.91 (0.51)	—	1.08 (1.04 to 1.13)	—
Collaring date of calf	1	4,017.27	4.54	0.06	0.56 (0.02)	24.06 (<0.01, *df* = 1)	2.11 (0.42)	—	1.09 (1.06 to 1.13)	—
Linear year	1	4,018.31	5.59	0.03	0.56 (0.02)	23.01 (<0.01, *df* = 1)	—	−0.92 (0.19)	—	0.91 (0.88 to 0.95)
Collaring date of calf + caribou herd (random)	2	4,018.99	6.27	0.02	—	24.34	2.04 (0.42)	—	1.09 (1.05 to 1.13)	—
Linear year + caribou herd (random)	2	4,020.05	7.32	0.01	—	23.29	—	−0.91 (0.19)	—	0.91 (0.88 to 0.95)
Year (random)	1	4,025.18	12.46	0.00	—	16.14	—	—	—	—
Caribou herd (random)	1	4,037.94	25.21	0.00	—	3.39	—	—	—	—
Null	0	4,039.32	26.59	0.00	—	—	—	—	—	—

^a^ Concordance index (c) was estimated from fixed‐effect models, and likelihood‐ratio test (LRT) comparing model with null model was estimated for fixed‐ and mixed‐effects models.

**TABLE E3:** Average AICc model weights (*w_i_*)^a^, adjusted conditional *R* ^2^ values^a^, and coefficient estimates for models of calf:cow ratio across the growth phase (*n* = 107 estimates of calf:cow ratios from 18 herds from 1979 to 1997) of woodland caribou (*Rangifer tarandus caribou*) population dynamics in Newfoundland, Canada

Model	*k*	*w_i_*	*R* ^2^	Linear year (1 year)	Survey date (30 days)	Herd size (100 caribou)
Linear year + survey date + caribou herd (random)	3	0.59	0.41	−0.0064 (*SE* = 0.0019; 95% CI = −0.0102 to −0.0027)	0.0968 (*SE* = 0.0218; 95% CI = 0.0541 to 0.1394)	—
Linear year + survey date + herd size + caribou herd (random)	4	0.34	0.42	−0.0056 (*SE* = 0.002; 95% CI = −0.0096 to −0.0017)	0.0981 (*SE* = 0.0217; 95% CI = 0.0556 to 0.1406)	−0.005 (*SE* = 0.0046; 95% CI = −0.0141 to 0.004)
Survey date + herd size + caribou herd (random)	3	0.05	0.29	—	0.1045 (*SE* = 0.0225; 95% CI = 0.0603 to 0.1487)	−0.0086 (*SE* = 0.0043; 95% CI = −0.0171 to −0.0001)
Survey date + caribou herd (random)	2	0.02	0.24	—	0.1033 (*SE* = 0.023; 95% CI = 0.0582 to 0.1484)	—
Linear year + caribou herd (random)	2	0.00	0.30	−0.0071 (*SE* = 0.0021; 95% CI = −0.0111 to −0.003)	—	—
Linear year + herd size + caribou herd (random)	3	0.00	0.30	−0.0065 (*SE* = 0.0022; 95% CI = −0.0108 to −0.0022)	—	−0.0039 (*SE* = 0.005; 95% CI = −0.0137 to 0.0059)
Herd size + caribou herd (random)	2	0.00	0.15	—	—	−0.0078 (SE = 0.0047; 95% CI = −0.017 to 0.0014)
Caribou herd (random)	1	0.00	0.09	—	—	—

^a^ These are average values as a result of the multiple imputation process. As such, these values have a measure of variance (*SE*). We excluded *SE* ≤ 0.01 for brevity.

**TABLE E4:** Average AICc model weights (*w_i_*)^a^, adjusted conditional *R* ^2^ values^a^, and coefficient estimates for models of calf:cow ratio across the declines phase (*n* = 142 estimates of calf:cow ratio from 24 herds from 2002 to 2014) of woodland caribou (*Rangifer tarandus caribou*) population dynamics in Newfoundland, Canada

Model	*k*	*w_i_*	*R* ^2^	Linear year (1 year)	Survey date (30 days)	Herd size (100 caribou)
Caribou herd (random)	1	0.20	0.31	—	—	—
Linear year + caribou herd (random)	2	0.19	0.33	−0.0027 (*SE* = 0.0019; 95% CI = −0.0065 to 0.001)	—	—
Survey date + caribou herd (random)	2	0.14	0.31	—	0.015 (*SE* = 0.0126; 95% CI = −0.0098 to 0.0397)	—
Linear year + herd size + caribou herd (random)	3	0.13	0.32	−0.0038 (*SE* = 0.0021; 95% CI = −0.0079 to 0.0003)	—	−0.0044 (*SE* = 0.0035; 95% CI = −0.0112 to 0.0024)
Linear year + survey date + caribou herd (random)	3	0.09	0.33	−0.0024 (*SE* = 0.002; 95% CI = −0.0062 to 0.0015)	0.0116 (*SE* = 0.0128; 95% CI = −0.0136 to 0.0368)	—
Linear year + survey date + herd size + caribou herd (random)	4	0.09	0.31	−0.0035 (*SE* = 0.0021; 95% CI = −0.0077 to 0.0006)	0.0155 (*SE* = 0.0131; 95% CI = −0.0101 to 0.0411)	−0.0053 (*SE* = 0.0035; 95% CI = −0.0122 to 0.0016)
Herd size + caribou herd (random)	2	0.08	0.30	—	—	−0.0019 (*SE* = 0.0032; 95% CI = −0.0081 to 0.0044)
Survey date + herd size + caribou herd (random)	3	0.07	0.30	—	0.0182 (*SE* = 0.0131; 95% CI = −0.0074 to 0.0439)	−0.0031 (*SE* = 0.0033; 95% CI = −0.0096 to 0.0033)

^a^ These are average values as a result of the multiple imputation process. As such, these values have a measure of variance (*SE*). We excluded *SE* ≤ 0.01 for brevity
